# Supplementary material for: Genetic risk predicts adolescent mood pathology via sexual differentiation of brain function and physiological aging
Source: Nat Commun. 2025 Jul 1;16:5593. doi: 10.1038/s41467-025-60686-5 (PMC12214705; doi:10.1038/s41467-025-60686-5)
Supplement: Supplementary file 1 — Supplementary Information [file 41467_2025_60686_MOESM1_ESM.pdf]

Supplementary Information

Supplemental Results/Figures

Figure S1. Results of the CCA (with 10-fold cross-validation) linking PhenoAge to BMI, financial deprivation and cause-specific unplanned medical visits.

Figure S2. The brain LV from the behavioral-PLS analysis linking the psychopathology and physiology variates from Analysis 1 (cf. Figure 3) to sexual differentiation in brain function in the ABCD sample (Gordon atlas).

Figure S3. The thresholded brain LV from the behavioral-PLS analyses linking the confound-denoised psychopathology and physiology variates from Analysis 1 (cf. Figure 3) to sexual differentiation in brain function in the ABCD sample.

Figure S4. The unthresholded brain LV from the behavioral-PLS analysis linking the confound-denoised psychopathology and physiology variates from Analysis 1 (cf. Figure 3) to sexual differentiation in brain function in the ABCD sample.

Figure S5. Serial mediational model testing the role of T1 sexual differentiation in brain function (cf Figure 4) and T2 physiology (cf. Figure 3-B) in mediating the impact of genetic risk for anxiety on rising depression and anxiety disorder-related symptoms (cf. Figure 3-A) in the ABCD sample for *p*-value of 10^{-4} (panel A), 10^{-5} and including the MHC region (panel B), as well as 10^{-4} including the MHC region (panel C) for the contributing anxiety SNPs (Schaefer atlas)

Figure S6. Serial mediational model testing the role of T1 sexual differentiation in brain function (cf Figure 4) and T2 physiology (cf. Figure 3-B) in mediating the impact of genetic risk for MDD on rising depression and anxiety disorder-related symptoms (cf. Figure 3-A)

in the ABCD sample for *p*-value of 5 x 10^{-8} (panel A), 5 x 10^{-8} and including the MHC region (panel B), as well as 10^{-5} including the MHC region (panel C) for the contributing MDD SNPs (Schaefer atlas)

Figure S7. Serial mediational model testing the role of T1 sexual differentiation in brain function (cf Figure 4) and T2 physiology (cf. Figure 3-B) in mediating the impact of genetic risk for ADHD on rising depression and anxiety disorder-related symptoms (cf. Figure 3-A) in the ABCD sample for *p*-value of 5 x 10^{-8} (panel A), 10^{-5} (panel B), as well as 10^{-4} (panel C) for the contributing ADHD SNPs (Schaefer atlas)

Figure S8. Serial mediational model testing the role of T1 sexual differentiation in brain function (cf Figure S2) and T2 physiology (cf. Figure 3-B) in mediating the impact of genetic risk for anxiety on rising depression and anxiety disorder-related symptoms (cf. Figure 3-A) in the ABCD sample for *p*-value of 10^{-5} (panel A), *p*-value of 10^{-5} and including the MHC region (panel B), *p*-value of 10^{-4} (panel C) as well as 10^{-4} including the MHC region (panel D) for the contributing anxiety SNPs (Gordon atlas)

Figure S9. The sexual differentiation brain LV from Analysis 2 linked by CCA to sex, individual differences in psychiatric disorder risk and maturation-relevant factors, as well as psychiatric disorder- and maturation-relevant neural sexual differentiation patterns in the HCP-D sample (Gordon atlas).

Figure S10. Residual QC-FC correlations and distance-dependence of the motion artifact after individual participant-level denoising

Supplemental Results/Figures

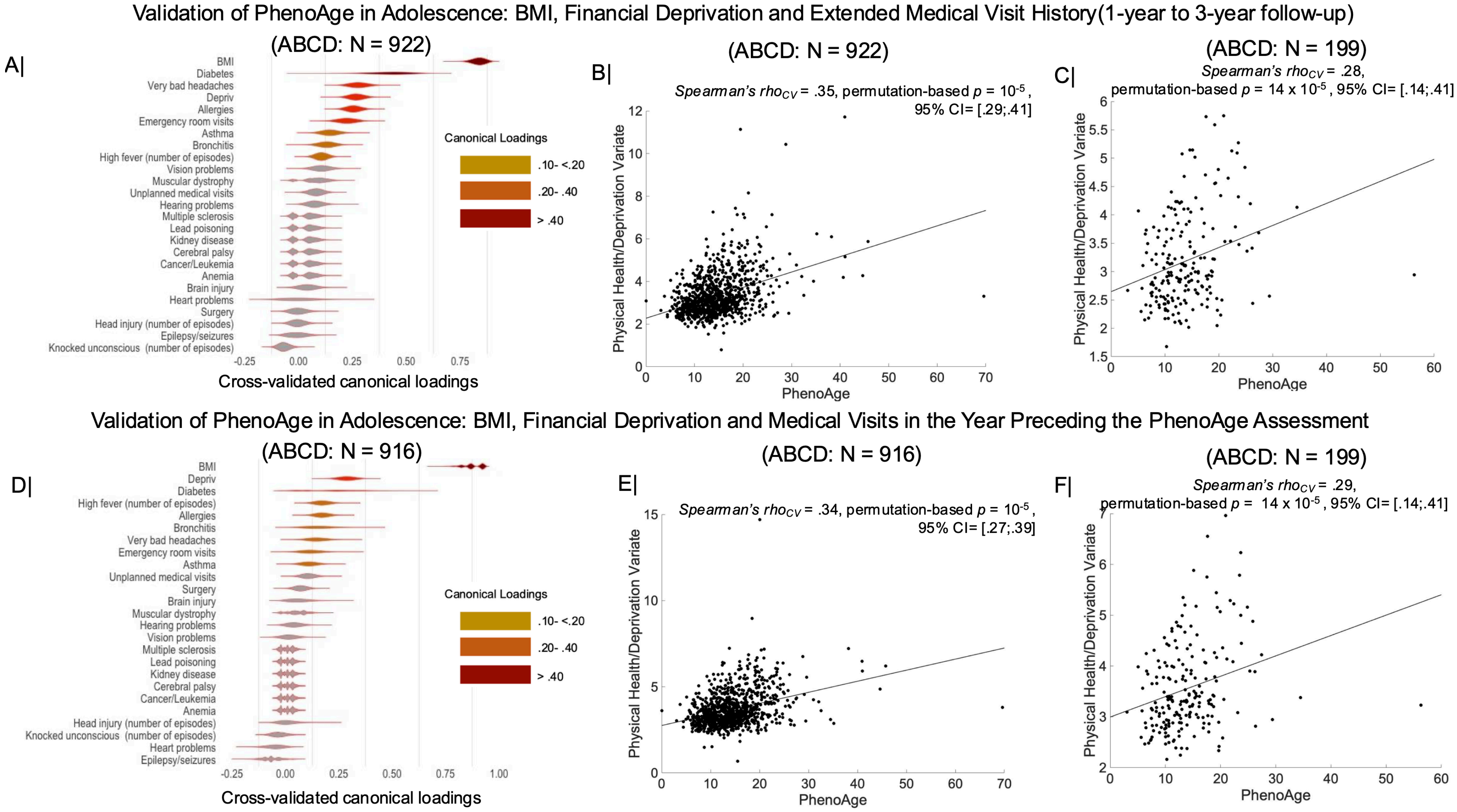

Figure S1. Results of the CCA (with 10-fold cross-validation) linking PhenoAge to BMI, financial deprivation and cause-specific unplanned

medical visits. Panels A-C depict results based on medical history data from the age of 10 to the age of 13 in the largest biologically unrelated ABCD sample with available data (N= 922, panels A, B) and our target ABCD sample (N = 199, panel C). Panels D-F depict results based on medical history data from the year preceding the collection of the PhenoAge blood chemistry data in the largest biologically unrelated ABCD sample with available data (N= 916, panels D, E) and our target ABCD sample (N = 199, panel F). BMI = body mass index. CCA = canonical correlation analysis. Depriv = financial deprivation.

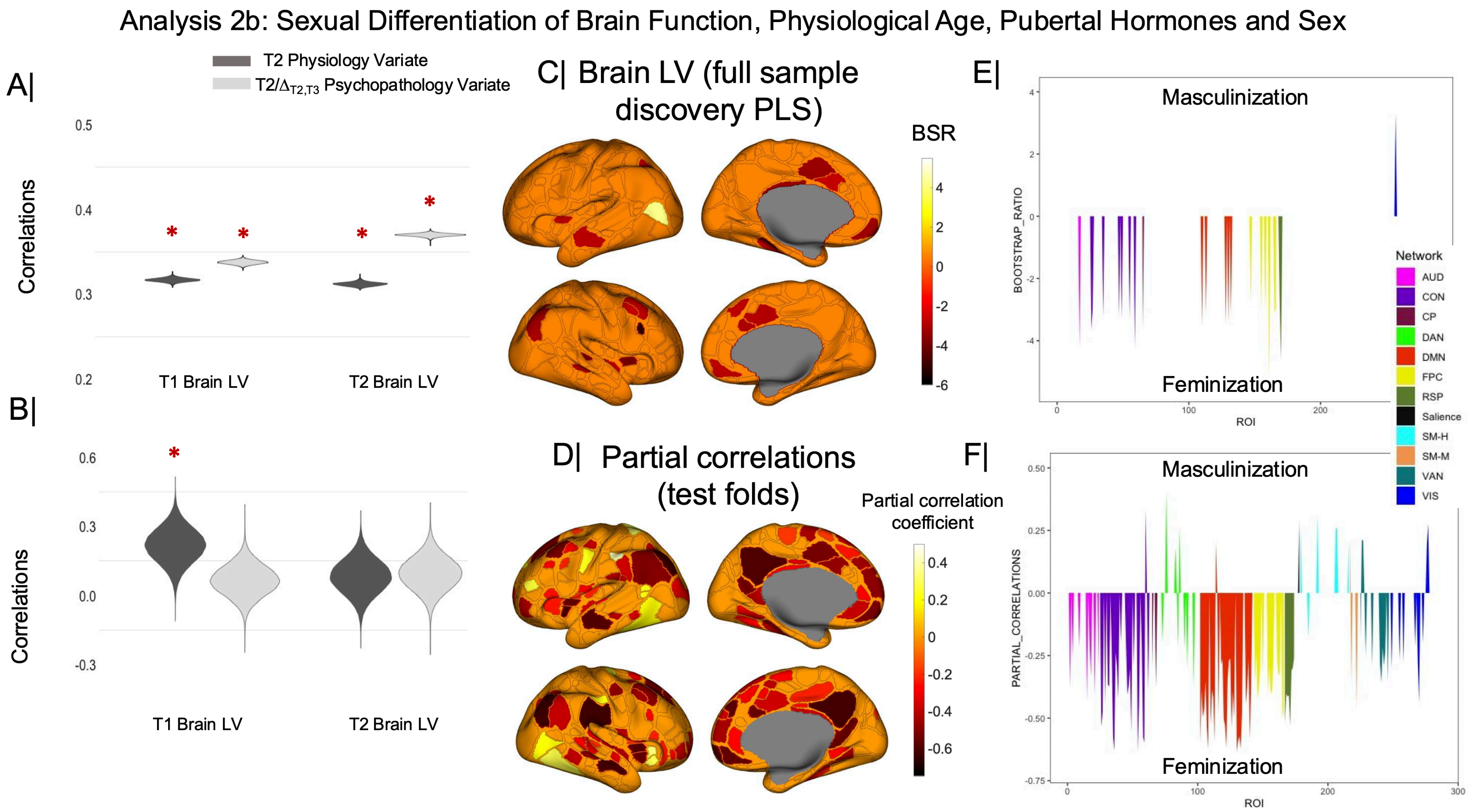

Figure S2. The brain LV from the behavioral-PLS analysis linking the psychopathology and physiology variates from CCA 1 (cf. Figure 3) to

neural sexual differentiation in the ABCD sample. Panel A shows the correlations of the two CCA 1 variates with the Time 1 and Time 2 brain LV scores in the discovery PLS analysis. Panel B shows the correlations of the two CCA 1 variates with the predicted Time 1 and Time 2 brain LV scores (based on the 10-fold cross-validation procedure). A red asterisk indicates a robust correlation between the respective CCA 1 variate and the discovery (panel A) or predicted (panel B) brain LV score across all participants. The violin plots in panels A and B depict the distribution of these correlation coefficients across the 100,000 bootstrap samples from the discovery (panel A) or cross-validated (panel B) PLS analysis. Panel C depicts the ROI-specific weights/loadings on the brain LV identified with the discovery PLS analysis with a bootstrap ratio greater than 2.75 in absolute value (equivalent to a 99% CI). Panel D depicts the Gordon ROIs robustly correlated (based on cross-validated 99% confidence intervals) with the predicted value of the brain LV from the cross-validation procedure. These are partial correlations controlling for the confounders listed under "Control variables". To facilitate interpretation, panels E and F present Gordon network-based distributions of PLS weights (panel E) or partial correlations (panel F) summarizing the ROI-specific results from panels (C) and (D), respectively. In panels C-F, positive values indicate masculinization, whereas negative values indicate feminization of the connectivity patterns associated with a specific ROI. As stated in the main text, sexual differentiation was estimated in reference to resting state connectivity data from the Human Connectome Project. T1 = Time 1. T2 = Time 2. PLS = partial least squares. LV= latent variable. BSR = bootstrap ratio. Gordon networks: AUD = auditory; CON = cingulo-opercular; CP = cingulo-parietal; DMN = default mode; DAN = dorsal attention; FPC = frontoparietal; RSP = retrosplenial; SAL = salience. SM-A = somatomotor-A; SM-B = somatomotor-B. VAN = ventral attention; VIS = visual.

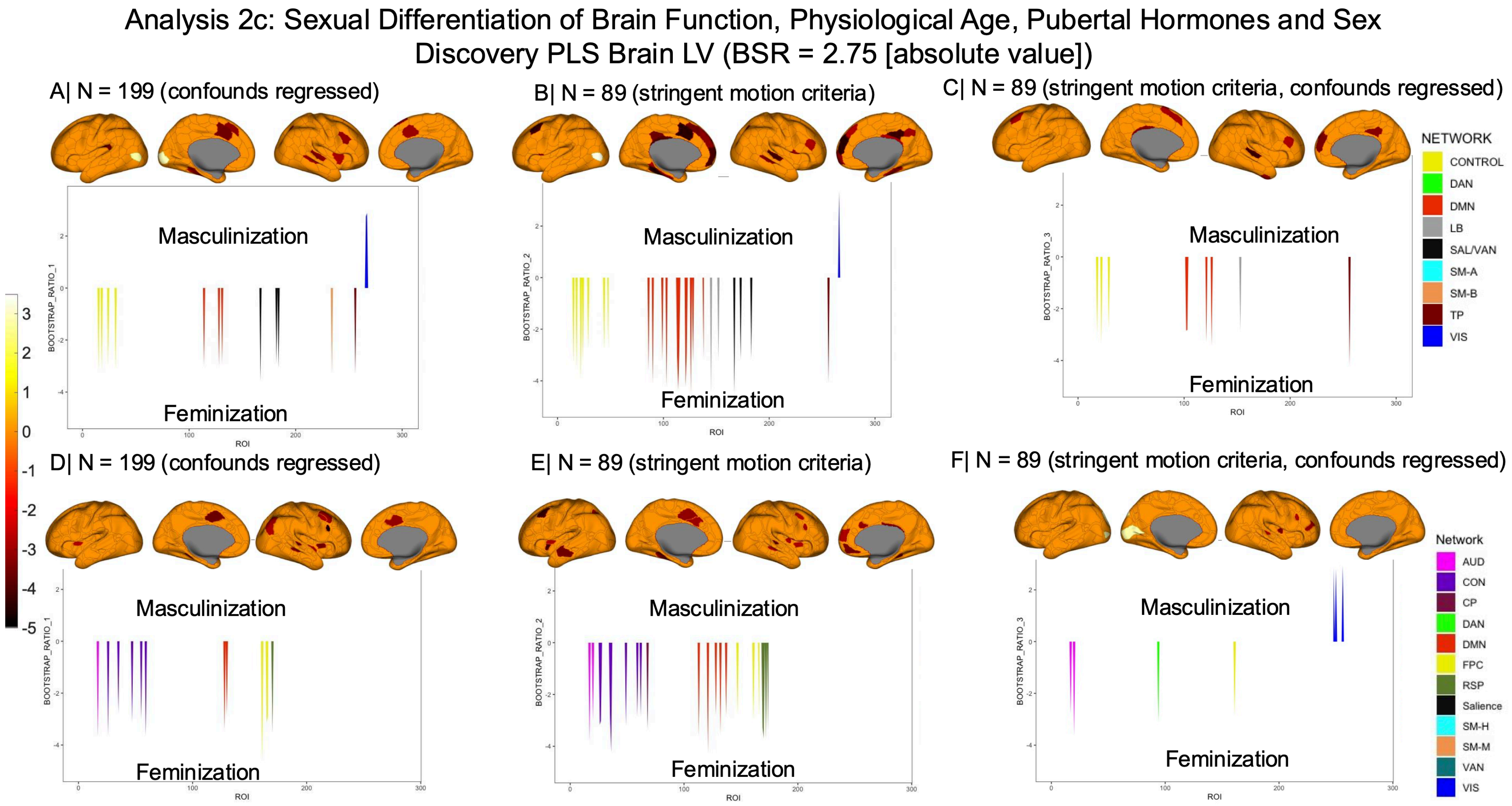

Figure S3. The thresholded brain LV from the behavioral-PLS analyses linking the confound-denoised psychopathology and physiology variates from Analysis 1 (cf. Figure 3) to sexual differentiation in brain function in the ABCD sample based on Schaefer atlas data (panels A-C) and the

Gordon atlas data (panels D-F). In all panels, the ROI weights have been thresholded based on a BSR greater than 2.75 in absolute value (equivalent to a 99% CI). BSR = bootstrap ratio. ROI = region-of-interest.

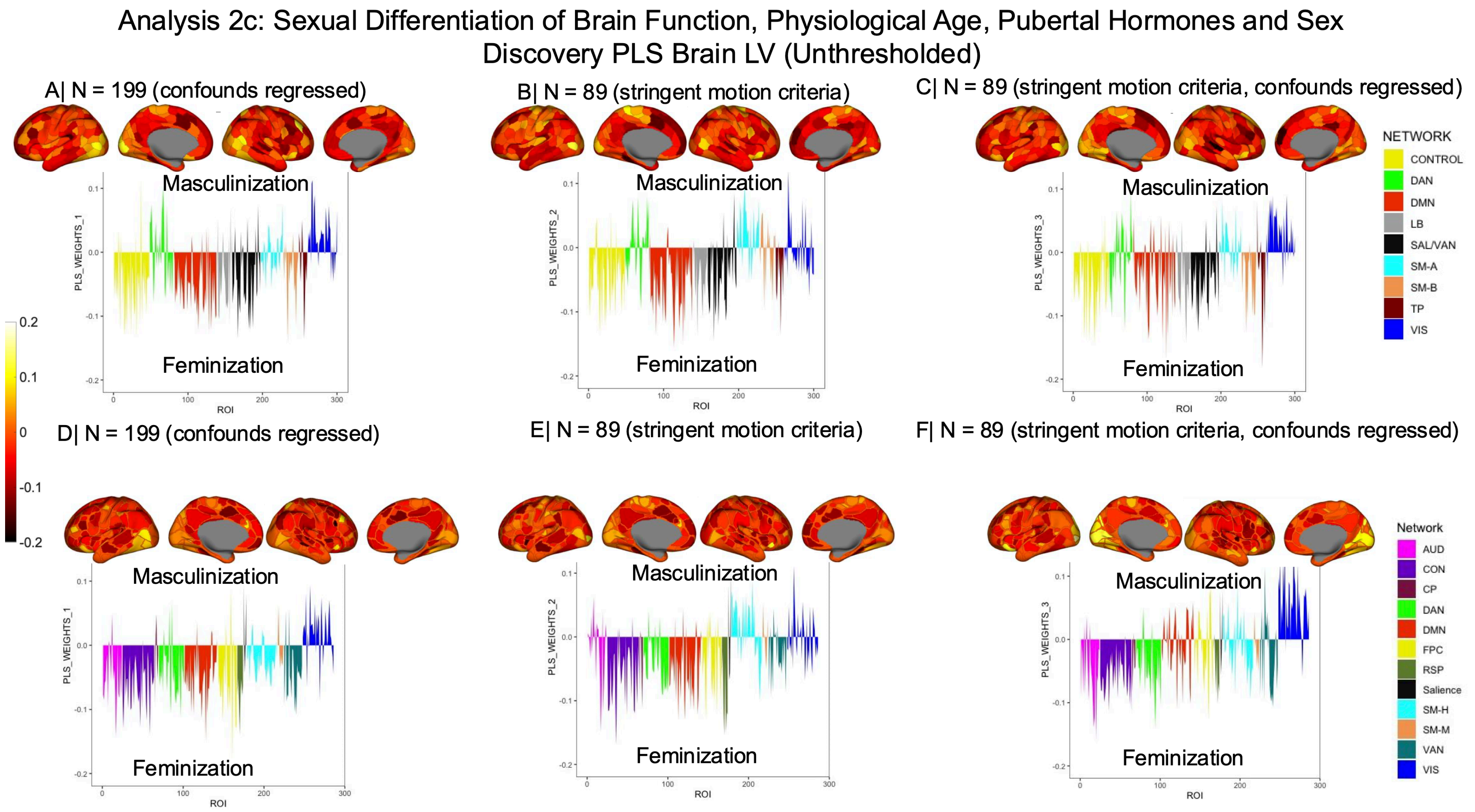

Figure S4. The unthresholded brain LV from the behavioral-PLS analysis linking the confound-denoised psychopathology and physiology

variates from Analysis 1 (cf. Figure 3) to sexual differentiation in brain function in the ABCD sample based on Schaefer atlas data (panels A-C) and the Gordon atlas data (panels D-F).

Analysis 3: Serial Mediation Models with Alternate *P*-values for the ANX PRS Contributing SNPs (Schaefer Atlas)

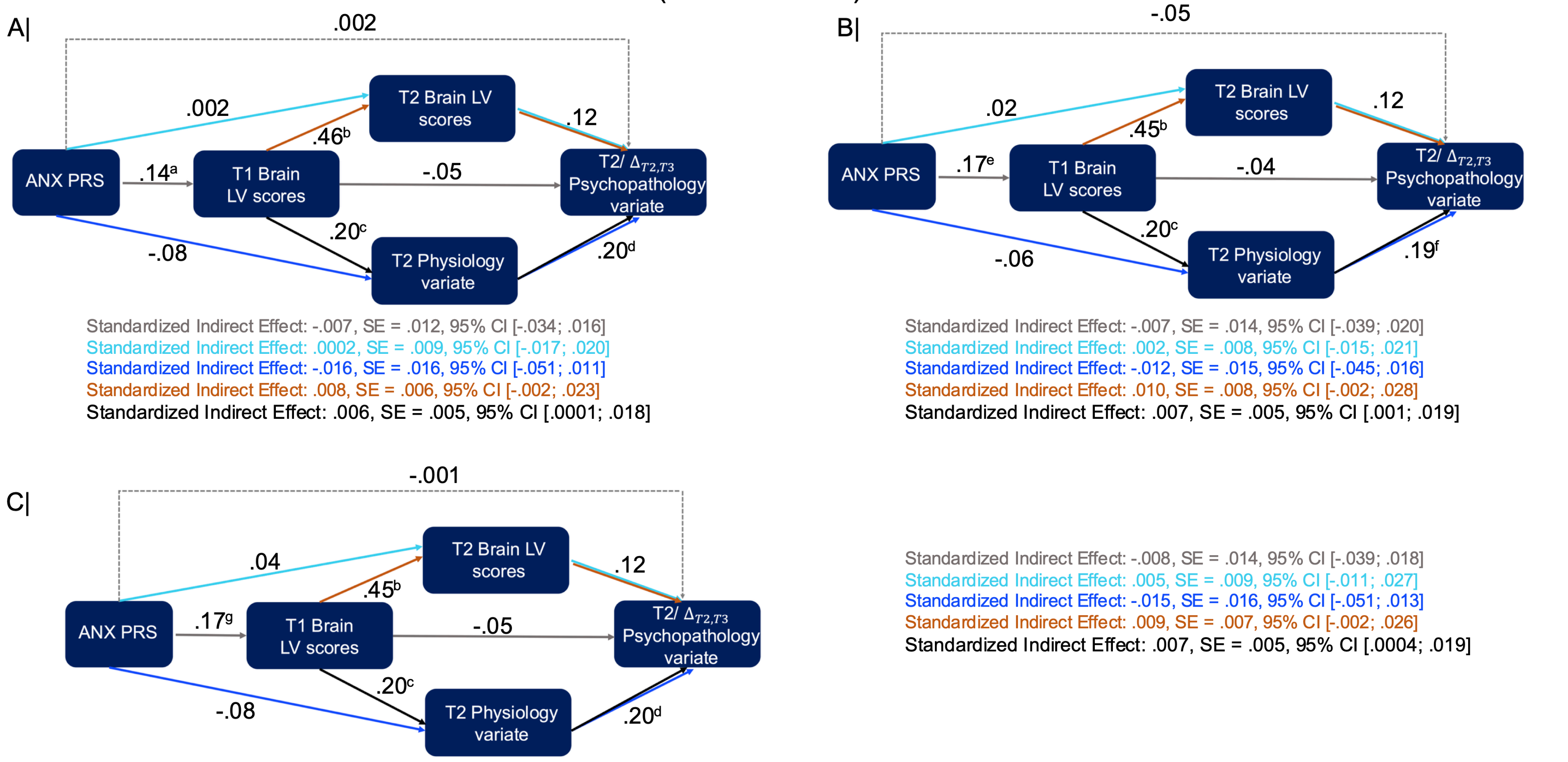

Figure S5. Serial mediational model testing the role of T1 sexual differentiation in brain function (cf Figure 4) and T2 physiology (cf. Figure 3-B) in mediating the impact of genetic risk for anxiety on rising depression and anxiety disorder-related symptoms (cf. Figure 3-A) in the ABCD sample for p-value of 10^{-4} (panel A), 10^{-5} and including the MHC region (panel B), as well as 10^{-4} including the MHC region (panel C) for the contributing anxiety SNPs (Schaefer atlas). N = 199 (ABCD youths). ANX = anxiety. LV = latent variable. MHC = major histocompatibility complex. PRS = polygenic risk score. SNP = single nucleotide polymorphism. T1 = Time 1. T2 = Time 2. T3 = Time 3.

95% confidence intervals for the indirect effects were estimated using percentile bootstrap with 50,000 bootstrap samples. All p-values of the t-statistic associated with each path are two-tailed. [a]$p = .036$ [degrees-of-freedom = 197]. [b]$p = 10^{-8}$ [degrees-of-freedom = 196]. [c]$p = .004$ [degrees-of-freedom = 196]. [d]$p = .011$ [degrees-of-freedom = 194]. [e]$p = .008$ [degrees-of-freedom = 197]. [f]$p = .013$ [degrees-of-freedom = 194]. [g]$p = .017$ [degrees-of-freedom = 197].

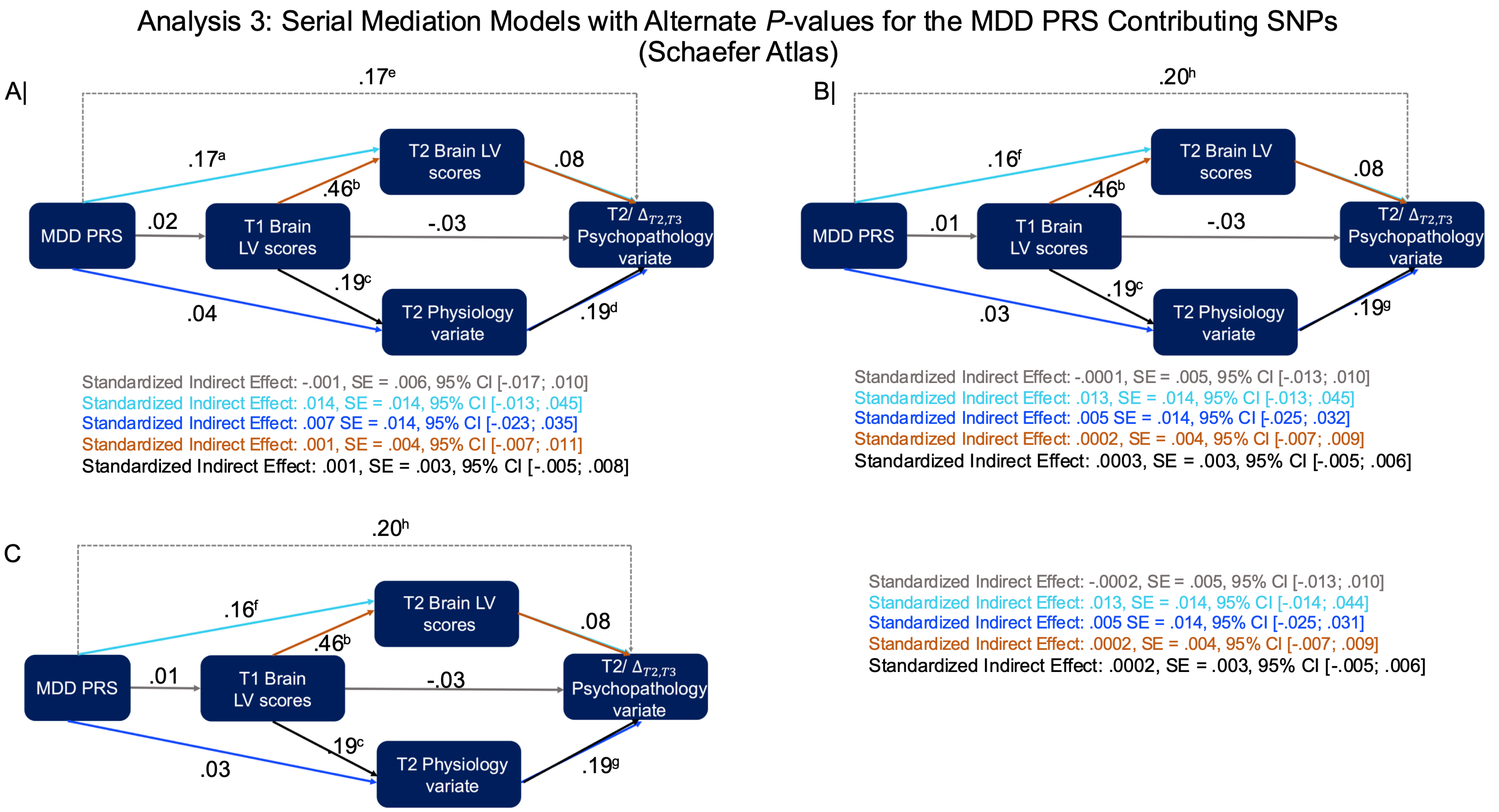

Figure S6. Serial mediational model testing the role of T1 sexual differentiation in brain function (cf Figure 4) and T2 physiology (cf. Figure 3-

B) in mediating the impact of genetic risk for MDD on rising depression and anxiety disorder-related symptoms (cf. Figure 3-A) in the ABCD sample for *p*-value of 5 x 10^{-8} (panel A), 5 x 10^{-8} and including the MHC region (panel B), as well as 10^{-5} including the MHC region (panel C) for the contributing MDD SNPs (Schaefer atlas). N = 199 (ABCD youths). LV = latent variable. MDD = major depressive disorder. MHC = major histocompatibility complex. PRS = polygenic risk score. SNP = single nucleotide polymorphism. T1 = Time 1. T2 = Time 2. T3 = Time 3. 95% confidence intervals for the indirect effects were estimated using percentile bootstrap with 50,000 bootstrap samples. All *p*-values of the *t*-statistic associated with each path are two-tailed. [a]$p = .008$ [degrees-of-freedom = 196]. [b]$p = 10^{-8}$ [degrees-of-freedom = 196]. [c]$p = .006$ [degrees-of-freedom = 196]. [d]$p = .018$ [degrees-of-freedom = 194]. [e]$p = .030$ [degrees-of-freedom = 194]. [f]$p = .010$ [degrees-of-freedom = 196]. [g]$p = .017$ [degrees-of-freedom = 194]. [h]$p = .015$ [degrees-of-freedom = 194].

Analysis 3: Serial Mediation Models with Alternate *P*-values for the ADHD PRS Contributing SNPs (Schaefer Atlas)

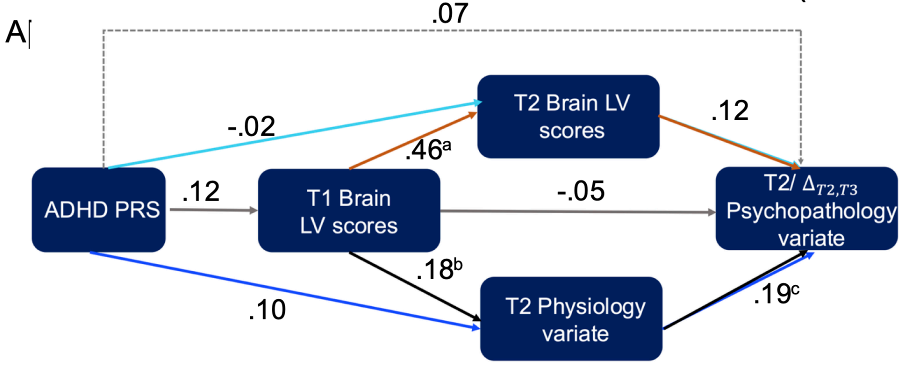

Standardized Indirect Effect: -.006, SE = .012, 95% CI [-.035; .012]
Standardized Indirect Effect: -.003, SE = .011, 95% CI [-.026; .020]
Standardized Indirect Effect: .019, SE = .015, 95% CI [-.005; .055]
Standardized Indirect Effect: .007, SE = .007, 95% CI [-.002; .023]
Standardized Indirect Effect: .004, SE = .004, 95% CI [-.001; .014]

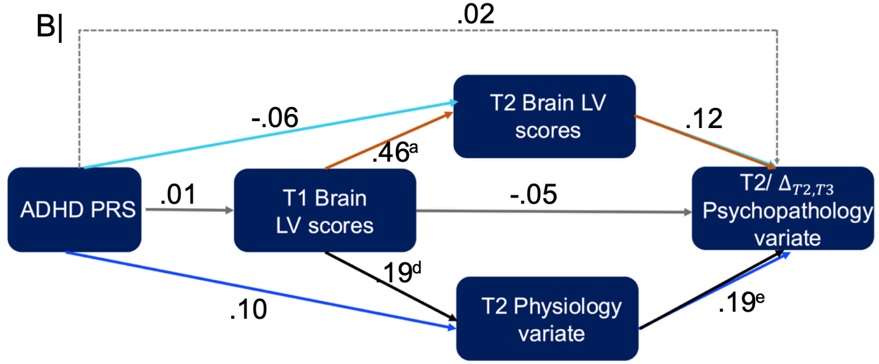

Standardized Indirect Effect: -.001, SE = .007, 95% CI [-.017; .012]
Standardized Indirect Effect: -.007, SE = .010, 95% CI [-.027; .013]
Standardized Indirect Effect: .019, SE = .016, 95% CI [-.007; .055]
Standardized Indirect Effect: .001, SE = .005, 95% CI [-.011; .011]
Standardized Indirect Effect: .0004, SE = .003, 95% CI [-.006; .007]

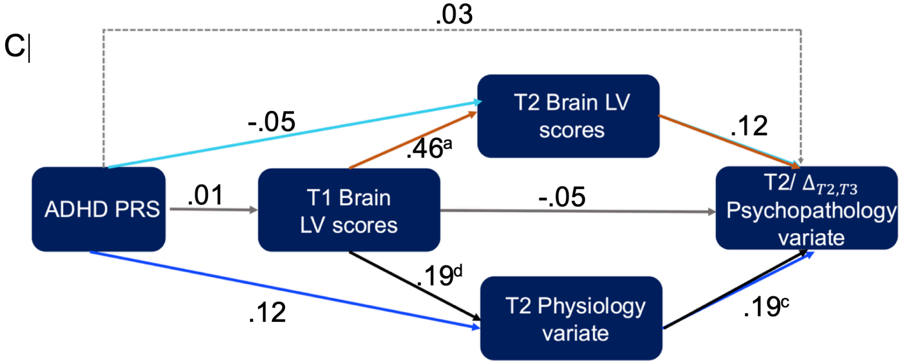

Standardized Indirect Effect: -.0004 SE = .006, 95% CI [-.016; .011]
Standardized Indirect Effect: -.006, SE = .009, 95% CI [-.025; .013]
Standardized Indirect Effect: .023, SE = .017, 95% CI [-.003; .061]
Standardized Indirect Effect: .001, SE = .005, 95% CI [-.010; .010]
Standardized Indirect Effect: .0003, SE = .003, 95% CI [-.005; .007]

Figure S7. Serial mediational model testing the role of T1 sexual differentiation in brain function (cf Figure 4) and T2 physiology (cf. Figure 3-B) in mediating the impact of genetic risk for ADHD on rising depression and anxiety disorder-related symptoms (cf. Figure 3-A) in the ABCD sample for *p*-value of 5 x 10^{-8} (panel A), 10^{-5} (panel B), as well as 10^{-4} (panel C) for the contributing ADHD SNPs (Schaefer atlas) N = 199 (ABCD youths). ADHD attention deficit hyperactivity disorder. LV = latent variable. PRS = polygenic risk score. SNP = single nucleotide polymorphism. T1 = Time 1. T2 = Time 2. T3 = Time 3. 95% confidence intervals for the indirect effects were estimated using percentile bootstrap with 50,000 bootstrap samples. All *p*-values of the *t*-statistic associated with each path are two-tailed. $^{a}p = 10^{-8}$ [degrees-of-freedom = 196]. $^{b}p = .009$ [degrees-of-freedom = 196]. $^{c}p = .013$ [degrees-of-freedom = 194]. $^{d}p = .006$ [degrees-of-freedom = 196]. $^{e}p = .012$ [degrees-of-freedom = 194].

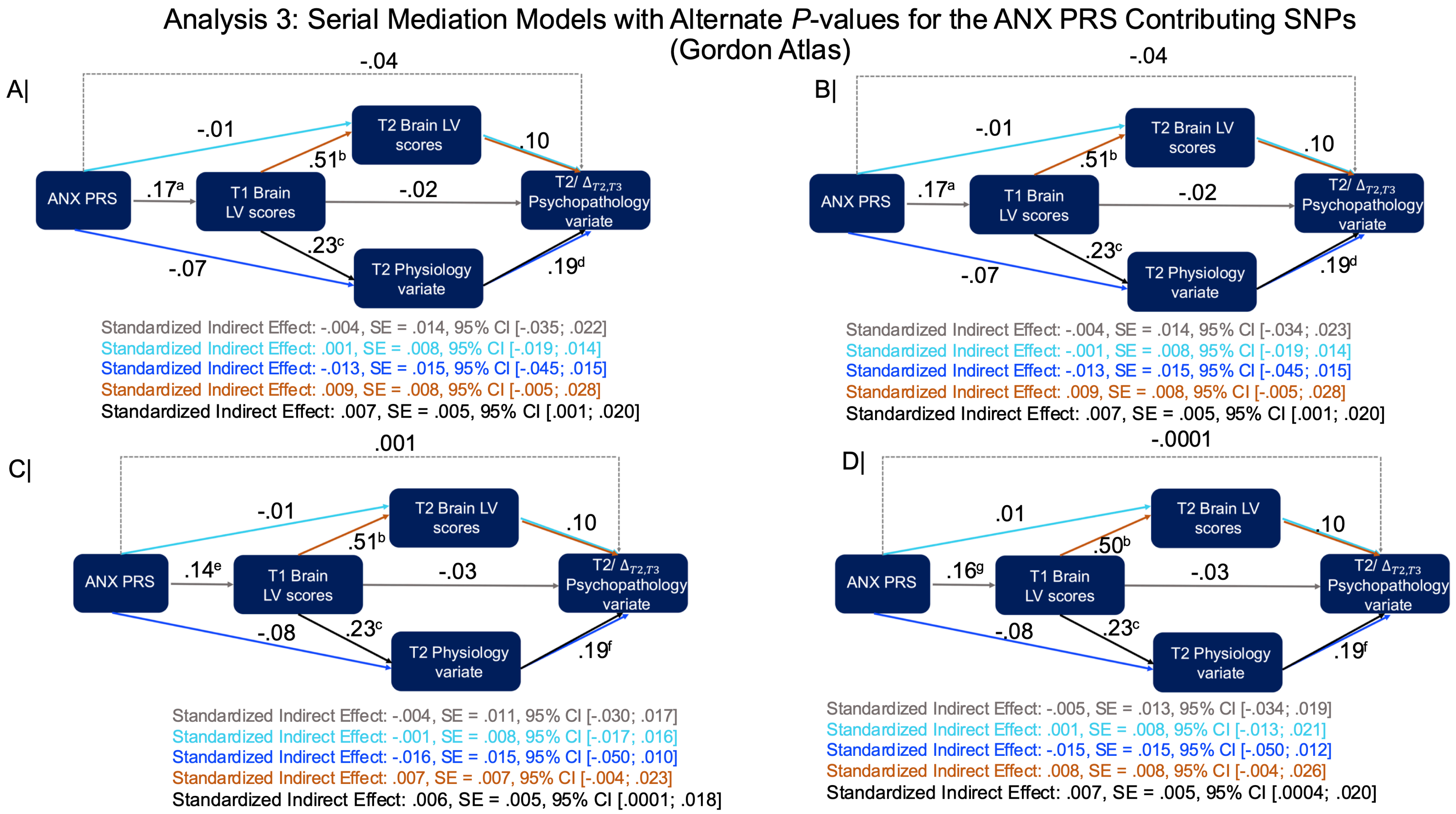

Figure S8. Serial mediational model testing the role of T1 sexual differentiation in brain function (cf Figure S2) and T2 physiology (cf. Figure 3-

B) in mediating the impact of genetic risk for anxiety on rising depression and anxiety disorder-related symptoms (cf. Figure 3-A) in the ABCD sample for *p*-value of 10^{-5} (panel A), *p*-value of 10^{-5} and including the MHC region (panel B), *p*-value of 10^{-4} (panel C) as well as 10^{-4} including the MHC region (panel D) for the contributing anxiety SNPs (Gordon atlas). N = 199 (ABCD youths). ANX = anxiety. LV = latent variable. MHC = major histocompatibility complex. PRS = polygenic risk score. SNP = single nucleotide polymorphism. T1 = Time 1. T2 = Time 2. T3 = Time 3. 95% confidence intervals for the indirect effects were estimated using percentile bootstrap with 50,000 bootstrap samples. All *p*-values of the *t*-statistic associated with each path are two-tailed. [a]$p = .009$ [degrees-of-freedom = 197]. [b]$p = 10^{-8}$ [degrees-of-freedom = 196]. [c]$p = .001$ [degrees-of-freedom = 196]. [d]$p = .015$ [degrees-of-freedom = 194]. [e]$p = .038$ [degrees-of-freedom = 197]. [f]$p = .013$ [degrees-of-freedom = 194]. [g]$p = .019$ [degrees-of-freedom = 197].

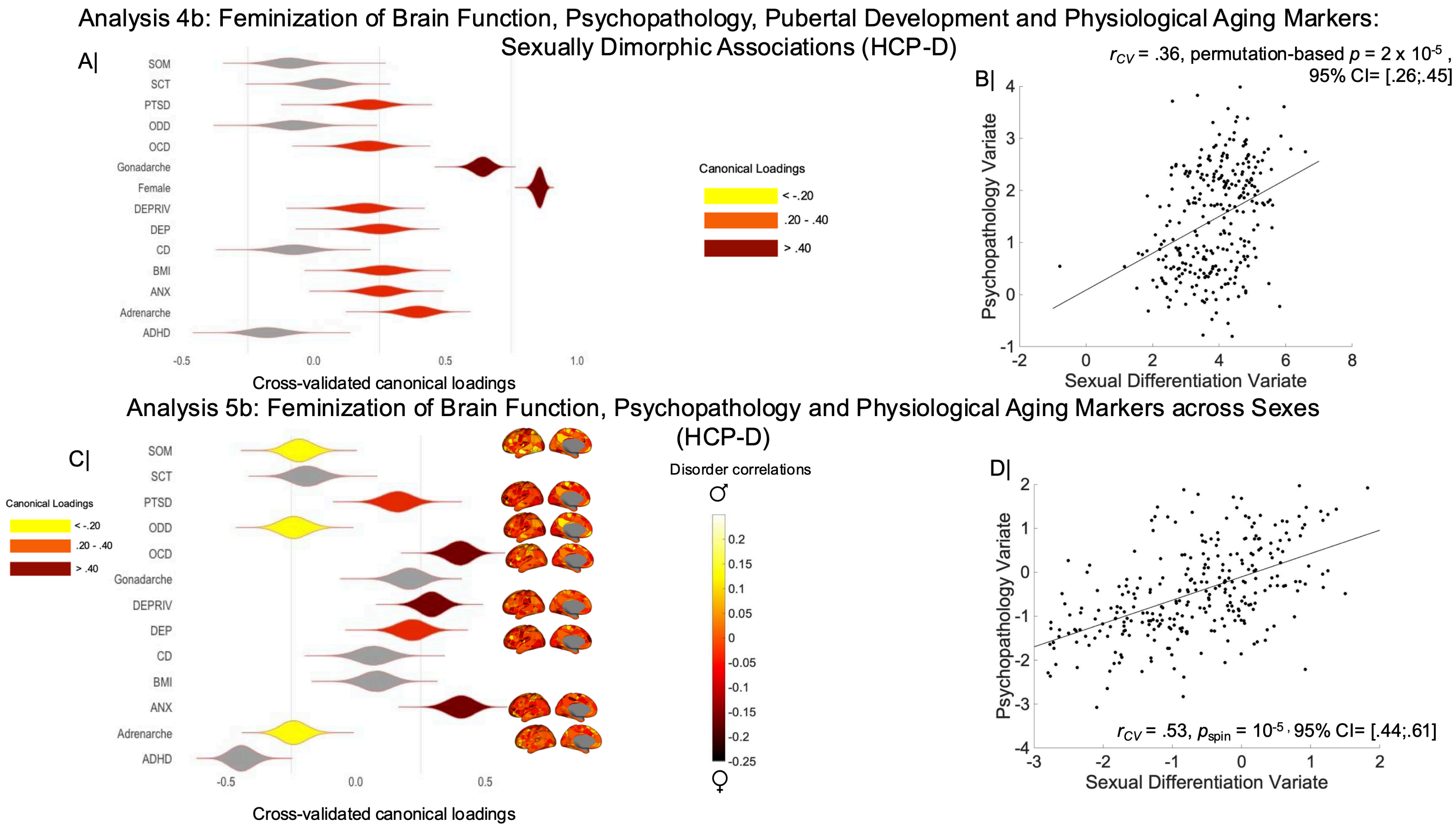

Figure S9. The sexual differentiation brain LV from Analysis 2 (N = 199) linked by CCA to sex, individual differences in psychiatric disorder

risk and maturation-relevant factors (panels A, B), as well as psychiatric disorder- and maturation-relevant neural sexual differentiation patterns in the HCP-D sample (panels C, D) (Gordon atlas). N = 277 (HCP-D). CCA = canonical correlation analysis. LV = latent variable.

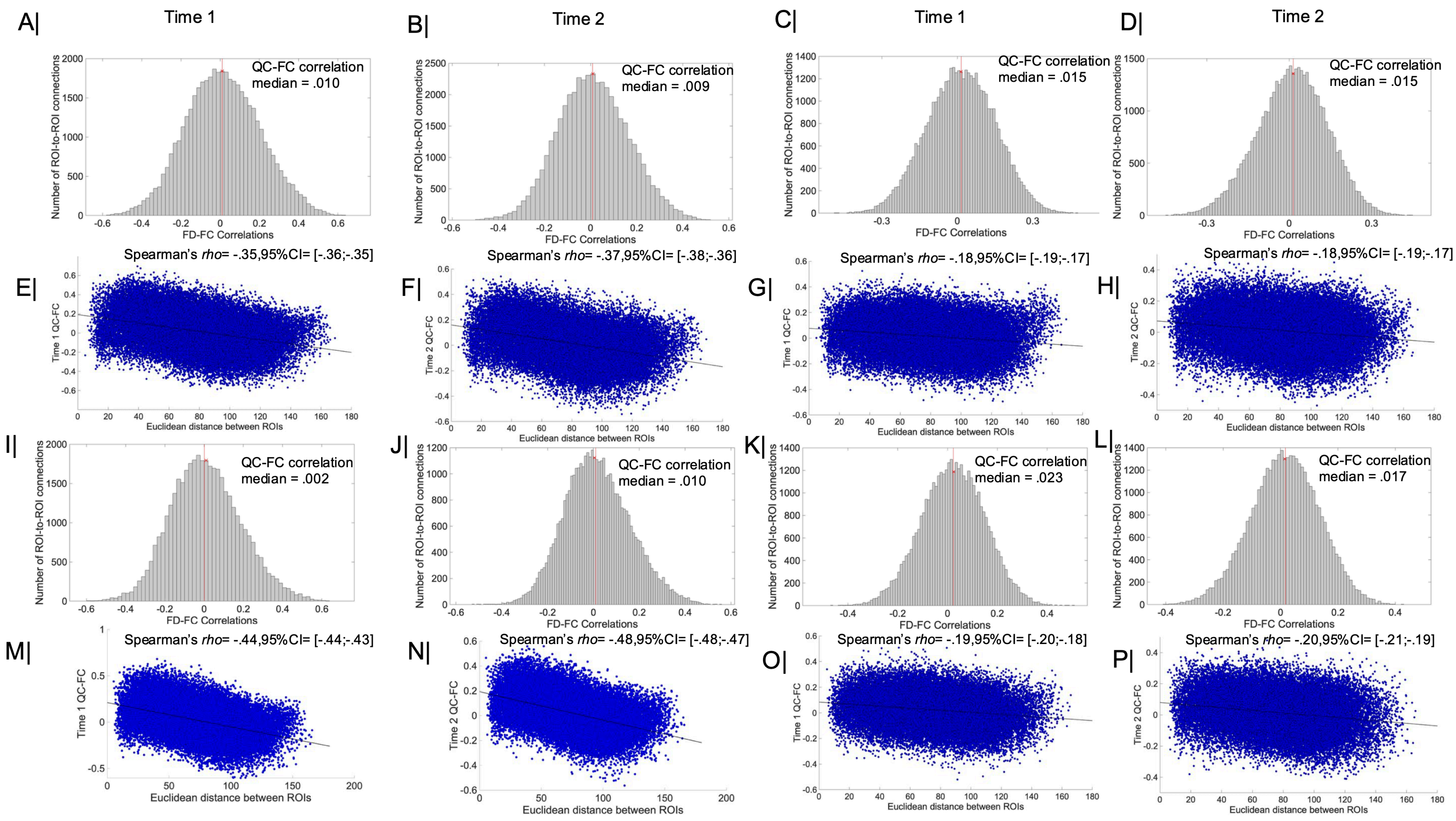
A|
Time 1
B|
Time 2
C|
Time 1
D|
Time 2
QC-FC correlation median = .010
QC-FC correlation median = .009
QC-FC correlation median = .015
QC-FC correlation median = .015
Number of ROI-to-ROI connections
FD-FC Correlations
Spearman's rho= -.35,95%CI= [-.36;-.35]
Spearman's rho= -.37,95%CI= [-.38;-.36]
Spearman's rho= -.18,95%CI= [-.19;-.17]
Spearman's rho= -.18,95%CI= [-.19;-.17]
E|
F|
G|
H|
Time 1 QC-FC
Time 2 QC-FC
Euclidean distance between ROIs
I|
J|
K|
L|
QC-FC correlation median = .002
QC-FC correlation median = .010
QC-FC correlation median = .023
QC-FC correlation median = .017
Spearman's rho= -.44,95%CI= [-.44;-.43]
Spearman's rho= -.48,95%CI= [-.48;-.47]
Spearman's rho= -.19,95%CI= [-.20;-.18]
Spearman's rho= -.20,95%CI= [-.21;-.19]
M|
N|
O|
P|

Figure S10. Residual QC-FC correlations and distance-dependence of the motion artifact after individual participant-level denoising. For the full study sample (N = 199), distribution of the QC-FC correlations is presented in panels A, B (Schaefer atlas) and I, J (Gordon atlas), while the association between QC-FC (cf panels A, B, I-J) and the Euclidean distance between the corresponding pair of ROIs is presented in panels E, F (Schaefer atlas) and M, N (Gordon atlas). For the low motion study sample (N = 89), distribution of the QC-FC correlations is presented in panels C, D (Schaefer atlas) and K, L (Gordon atlas), while the association between QC-FC (cf panels C, D, K, L) and the Euclidean distance between the corresponding pair of ROIs is presented in panels G, H (Schaefer atlas) and O, P (Gordon atlas). QC = quality control (i.e., average framewise displacement per participant). FC = functional connectivity.
