# Supplementary material for: Genetic risk predicts adolescent mood pathology via sexual differentiation of brain function and physiological aging
Source: Nat Commun. 2025 Jul 1;16:5593. doi: 10.1038/s41467-025-60686-5 (PMC12214705; doi:10.1038/s41467-025-60686-5)
Supplement: Supplementary file 2 — Reporting Summary [file 41467_2025_60686_MOESM2_ESM.pdf]

nature portfolio

Corresponding author(s): Raluca Petrican

Last updated by author(s): May 14, 2025

Reporting Summary

Nature Portfolio wishes to improve the reproducibility of the work that we publish. This form provides structure for consistency and transparency in reporting. For further information on Nature Portfolio policies, see our Editorial Policies and the Editorial Policy Checklist.

Statistics

For all statistical analyses, confirm that the following items are present in the figure legend, table legend, main text, or Methods section.

n/a	Confirmed	
☐	☒	The exact sample size (*n*) for each experimental group/condition, given as a discrete number and unit of measurement
☐	☒	A statement on whether measurements were taken from distinct samples or whether the same sample was measured repeatedly
☐	☒	The statistical test(s) used AND whether they are one- or two-sided<br>*Only common tests should be described solely by name; describe more complex techniques in the Methods section.*
☐	☒	A description of all covariates tested
☒	☐	A description of any assumptions or corrections, such as tests of normality and adjustment for multiple comparisons
☐	☒	A full description of the statistical parameters including central tendency (e.g. means) or other basic estimates (e.g. regression coefficient) AND variation (e.g. standard deviation) or associated estimates of uncertainty (e.g. confidence intervals)
☐	☒	For null hypothesis testing, the test statistic (e.g. *F*, *t*, *r*) with confidence intervals, effect sizes, degrees of freedom and *P* value noted<br>*Give P values as exact values whenever suitable.*
☒	☐	For Bayesian analysis, information on the choice of priors and Markov chain Monte Carlo settings
☒	☐	For hierarchical and complex designs, identification of the appropriate level for tests and full reporting of outcomes
☐	☒	Estimates of effect sizes (e.g. Cohen's *d*, Pearson's *r*), indicating how they were calculated

Our web collection on statistics for biologists contains articles on many of the points above.

Software and code

Policy information about availability of computer code

Data collection	We did not use any software to collect the data as we analysed already collected data.
Data analysis	Analyses were conducted in Matlab (v24.10), Python3, SPSS (v 29.0). Images were created with functions from R package (v4.3.2). We used the following freely available toolboxes: PLINK 1.9 (https://zzz.bwh.harvard.edu/plink/), BioAge R (https://github.com/dayoonkwon/BioAge), Neuromaps (https://netneurolab.github.io/neuromaps/usage.html), FSL (v6.01), Permutation Analysis of Linear Models software package (PALM alpha116, https://github.com/andersonwinkler/PALM), the Connectome Workbench (https://www.humanconnectome.org/software/get-connectome-workbench), PLS (https://github.com/McIntosh-Lab/PLS/), BrainEigenModes(https://github.com/BMHLab/BrainEigenmodes_legacy) and PROCESS 4.2 macro for SPSS https://www.processmacro.org/download.html. Custom scripts were used to generate the spatial null maps used in the cross-validation of the multivariate models https://github.com/frantisekvasa/rotate_parcellation/commit/bb8b0ef10980f162793cc180cef371e83655c505 . Minimally preprocessed ABCD data had been run through the Multimodal Processing Stream, which combines FSL, Python, MATLAB, AFNI (https://afni.nimh.nih.gov/download) and Freesurfer (https://surfer.nmr.mgh.harvard.edu/fswiki/DownloadAndInstall) functions.

For manuscripts utilizing custom algorithms or software that are central to the research but not yet described in published literature, software must be made available to editors and reviewers. We strongly encourage code deposition in a community repository (e.g. GitHub). See the Nature Portfolio guidelines for submitting code & software for further information.

April 2023

Data

Policy information about availability of data

All manuscripts must include a data availability statement. This statement should provide the following information, where applicable:

- Accession codes, unique identifiers, or web links for publicly available datasets
- A description of any restrictions on data availability
- For clinical datasets or third party data, please ensure that the statement adheres to our policy

The raw data used in this report is available to researchers working at institutions recognized by NIMH at https://nda.nih.gov/abcd (ABCD) and at https://nda.nih.gov/ccf/lifespan-studies (HCP-D) following completion of the relevant data-use agreements. Researchers with different affiliations need to complete separate data-use agreements, signed by an authorized signing official from their respective institutions prior to submission to the NDA. Researchers need to create an account on https://nda.nih.gov/. Access is typically granted within a month from submitting the data-access request via the NDA site. Access to these data is controlled due to the highly sensitive nature of the data. Researchers need to apply for data access renewal on a yearly basis. Renewal requests need to include a progress report and are reviewed by the data access certification team. The ABCD data repository grows and changes over time. The ABCD data used in this report came from Data Release 5.1 (DOI: http://dx.doi.org/10.15154/z563-zd24). The HCP-D data used in this report came from Data Release 2.0 (DOI: 10.15154/1520708). With regard to brain atlases, the Schaefer atlas can be downloaded from https://github.com/ThomasYeoLab/CBIG and the Gordon atlas can be downloaded from https://wustl.app.box.com/v/parcels-release. The specific dataset (ABCD, HCP-D) used in this report can be accessed via the NDA site at 10.15154/te4p-qr97.708).

Research involving human participants, their data, or biological material

Policy information about studies with human participants or human data. See also policy information about sex, gender (identity/presentation), and sexual orientation and race, ethnicity and racism.

Reporting on sex and gender

All analyses included both male and female individuals, based on parental reports (ABCD/HCP-D) and self-reports (HCP). All analyses, apart from one (see Figure 2: analysis 5) tested for sex differences. In analysis 5 (see Figure 2), sex was controlled for.

Reporting on race, ethnicity, or other socially relevant groupings

For the ABCD and HCP-D participants, we used parental reports of the participants' race. For the HCP participants, we used their self-reported race. No other socially relevant groupings are reported in our manuscript.

In order to maximize interpretability of the model estimates, the discovery CCA (Analysis 1, Analysis 2) and PLS analyses were conducted on data that had not been residualized for the confounders listed below. To demonstrate the robustness of our results, the CCA and PLS cross-validation, as well as the mediation analyses controlled though for the following variables which were of no interest in the present study: (1) chronological age (for ABCD, at each wave contributing to the respective analysis) to ensure the reported inter-relationships hold irrespective of the participants' chronological age; (2) handedness (coded as "0" for right-handedness and "1" for non-right-handedness) to control for potential differences in the lateralization of the observed effects; (3) scanner site to account for scanner-related differences; (4) race because the genetic architecture of some risk loci may show some racial variations and to ensure that any associations with psychopathology hold irrespective of racial background, (5) adoption status (dummy coded "0"/"1" for the 3 ABCD adoptees and the 5 HCP-D adoptees), (6) (ABCD sample only) sex assigned at birth not corroborated by menstruation history at ages 11-12 or SNP analysis (dummy-coded 0"/"1" for 2 youths) , as well as (7) average modality-specific (i.e., resting state) motion per participant because it can adversely impact functional connectivity metrics.

The partial correlation analyses which identified patterns of neural sexual differentiation tracking variations in psychiatric disorder symptoms and physiological maturation (reported under "Psychiatric disorder-related maps of sexual differentiation in brain function") in the HCP-D sample controlled for biological sex in addition to the confounders listed above.

Population characteristics

ABCD: 199 adolescents (88 biological females), 9-13 years of age, 152 predominantly right-handed, identified by their parents as 3% Native American Indian, 1% Asian Indian, 13.6% Black, 1% Chinese, 2% Filipino/Filipina, .5% Japanese, .5% Korean, .5% Pacific Islander, .5% Vietnamese, 6.5% other or not reported racial background,82.4% White
HCP-D: 277 adolescents, 145 biological females, 250 predominantly right-handed, 8-17 years of age, identified by their parents as 0.4% American Indian/Alaska Native, 4.7% Asian, 8.3% Black, 18.4% mixed race, 1.4% other or not reported racial background, 66.8% White.
HCP: 336 young adults, 159 biological females, 300 predominantly right-handed, who self-identified as 7.7% Asian, 13.1% Black, 3.3 % more than one race, 73.5% White and 2.4% not reported.

Recruitment

Described in Garavan et al., 2018 (ABCD), Somerville et al., 2018 (HCP-D) or on the study website (HCP) https://www.humanconnectome.org/study/hcp-young-adult/project-protocol/recruitment

Ethics oversight

The study was approved by the local ethics committee of each dataset, and written informed consent was obtained from each participant or their authorized legal representative/guardian (the latter for ABCD and HCP-D youth) . For the ABCD study, the research ethics boards were Children's Hospital Los Angeles, Florida International University, Laureate Institute for Brain Research, Medical University of South Carolina, Oregon Health & Science University, SRI International, University of California San Diego, University of California Los Angeles, University of Colorado Boulder, University of Florida, University of Maryland at Baltimore, University of Michigan, University of Minnesota, University of Pittsburgh, University of Rochester, University of Utah, University of Vermont, University of Wisconsin-Milwaukee, Virginia Commonwealth University, Washington University in St. Louis and Yale university. For the HCP-D study, the ethics boards were Harvard University, University of California Los Angeles, University of Minnesota and Washington University in St. Louis. For the HCP study, the ethics board was Washington University in St. Louis.

Note that full information on the approval of the study protocol must also be provided in the manuscript.

Field-specific reporting

Please select the one below that is the best fit for your research. If you are not sure, read the appropriate sections before making your selection.

☒ Life sciences ☐ Behavioural & social sciences ☐ Ecological, evolutionary & environmental sciences

For a reference copy of the document with all sections, see nature.com/documents/nr-reporting-summary-flat.pdf

Life sciences study design

All studies must disclose on these points even when the disclosure is negative.

Sample size	We used the maximum number of participants with complete data on all variables of interest. For the ABCD and HCP-D samples, we used only the data recommended for inclusion by the respective study teams. This information is available in all the data packages available for download. For the HCP sample, we downloaded the preprocessed resting state data that was flagged as having passed the overall quality checks.
Data exclusions	Exclusion criteria were applied only to the HCP data. Specifically, we only included individuals aged 22-30 years (22-25 years: N = 141, 63 females; 26-30 years: N = 195, 96 females) in light of evidence (cited in the main text) that neurocognitive performance starts showing subtle signs of decline following the age of 30.
Replication	To test the generalizability of our models, we used ten-fold cross-validation for our partial least squares and canonical correlation analyses. All the replication attempts were successful.
Randomization	This is an observational study which contains no experimental conditions that may require random assignment of participants to groups.
Blinding	This is an observational study which contains no experimental conditions (hence no need for blinding).

Reporting for specific materials, systems and methods

We require information from authors about some types of materials, experimental systems and methods used in many studies. Here, indicate whether each material, system or method listed is relevant to your study. If you are not sure if a list item applies to your research, read the appropriate section before selecting a response.

Materials & experimental systems

n/a	Involved in the study	
☒	☐	Antibodies
☒	☐	Eukaryotic cell lines
☒	☐	Palaeontology and archaeology
☒	☐	Animals and other organisms
☒	☐	Clinical data
☒	☐	Dual use research of concern
☒	☐	Plants

Methods

n/a	Involved in the study	
☒	☐	ChIP-seq
☒	☐	Flow cytometry
☐	☒	MRI-based neuroimaging

Plants

Seed stocks	*Report on the source of all seed stocks or other plant material used. If applicable, state the seed stock centre and catalogue number. If plant specimens were collected from the field, describe the collection location, date and sampling procedures.*
Novel plant genotypes	*Describe the methods by which all novel plant genotypes were produced. This includes those generated by transgenic approaches, gene editing, chemical/radiation-based mutagenesis and hybridization. For transgenic lines, describe the transformation method, the number of independent lines analyzed and the generation upon which experiments were performed. For gene-edited lines, describe the editor used, the endogenous sequence targeted for editing, the targeting guide RNA sequence (if applicable) and how the editor was applied.*
Authentication	*Describe any authentication procedures for each seed stock used or novel genotype generated. Describe any experiments used to assess the effect of a mutation and, where applicable, how potential secondary effects (e.g. second site T-DNA insertions, mosiacism, off-target gene editing) were examined.*

Magnetic resonance imaging

Experimental design

Design type	resting state fMRI
Design specifications	ABCD<br>Four resting state fMRI scans (eyes open with passive crosshair viewing), lasting 20 minutes in total, were collected at each available time point (baseline/two-year follow-up). Due to data availability/quality, we used only 3 scans from each time point (6 resting state scans in total).<br><br>HCP-D<br>Four resting state fMRI scans (eyes open with passive crosshair viewing), lasting approximately 25 minutes<br><br>HCP<br>Four resting state fMRI scans (eyes open with passive crosshair viewing), lasting approximately 60 minutes<br><br>In each sample, the ROI-to-ROI correlations were run separately for each run (417 volumes for HCP; 375 volumes for ABCD/HCP-D) and the resulting ROI-to-ROI (300 x 300) matrices were averaged across the 3 (ABCD) or 4 (HCP/HCP-D) runs available at each time point. Thus, across the three samples (HCP, ABCD, HCP-D), ROI-to-ROI functional connectivity patterns were estimated across the same duration (~ 5 minutes/run, averaged across 3 runs at each time point for the ABCD data and across 4 runs at the only available timepoint for the HCP and HCP-D participants).
Behavioral performance measures	No behavioural performance measures were collected.

Acquisition

Imaging type(s)	resting state fMRI
Field strength	3T
Sequence & imaging parameters	ABCD<br>The fMRI data were acquired with a multiband EPI sequence (TR=800 ms, TE=30 ms, flip angle=52°, FOV = 216 x 216 mm, 60 slices of 2.4 × 2.4 mm in-plane resolution, 2.4 mm thick, multiband acceleration factor of 6).<br><br>HCP-D<br>The fMRI data were acquired with a multiband gradient-recalled EPI sequence (TR = 800 ms, TE = 37 ms, flip angle = 52°, FOV = 208 mm, 104 × 90 matrix, 72 oblique axial slices, 2 mm isotropic voxels, multiband acceleration factor of 8).<br><br>HCP<br>Participants were scanned with a customized Siemens 3T “Connectome Skyra” scanner housed at Washington University in St. Louis. Functional images were acquired with a multiband EPI sequence (TR= 720 ms,TE= 33.1ms,flip angle= 52°, FOV= 208 mm,104 x 90 matrix, 72 slices of 2 x 2 mm in-plane resolution, 2 mm thick, no gap; multiband acceleration factor of 8). Two runs were acquired with a left-to-right (LR) and the other two with a right-to-left (RL) phase encoding sequence
Area of acquisition	whole-brain

Diffusion MRI ☐ Used ☒ Not used

Preprocessing

Preprocessing software	ABCD<br>Analyses were conducted on minimally preprocessed resting state fMRI data, which was available as part of the ABCD Study Curated Annual Release 5.0. Using the Multimodal Processing Stream, developed by the ABCD team, these data had been corrected for head motion, spatial and gradient distortions, bias field removal, and the “cleaned” functional images had been co-registered to the participant’s T1– weighted structural image. Using FSL and Matlab, we further applied the following steps: (1) elimination of initial volumes (8 volumes [Siemens, Philips], 5 volumes [GE DV25], 16 volumes [GE DV26] to allow the MR signal to reach steady state equilibrium, (2) linear regression-based removal of the mean time courses of cerebral white matter (WM), gray matter (GM), cerebrospinal fluid (CSF), as well as the quadratic trends and 24 motion terms (i.e., the six motion parameters, their first derivatives, and squares from the time course of each parcel. Prior to being regressed, the motion terms had been filtered to eliminate signals within the respiratory effect range (i.e., .31-.43 Hz)<br><br>HCP-D/HCP<br>Data were processed by applying the Generic fMRI Volume and Surface Processing Pipelines, multi-run independent component analysis (ICA) FIX denoising and multimodal surface matching registration. The Generic Volume Processing Pipeline accomplished removal of spatial and gradient distortions, correction for participant movement, bias field removal, spatial normalization to the standard Montreal Neurological Institute (MNI)-152 template (2 mm isotropic voxels), intensity normalization to a global mean and masking out of non-brain voxels. Subsequent temporal preprocessing steps involved weak high-pass temporal filtering with the goal of removing linear trends in the data. The Generic Surface Processing Pipeline

registered the functional data into a standard grayordinate space by projecting the cortical gray matter into a registered surface mesh with a standard number of vertices (32k_fs_LR mesh) and projecting the subcortical data to a set of subcortical gray matter voxels. A small amount of spatial smoothing (2 mm full-width-at-half-maximum [FWHM]) was also applied to the functional data at this step. The ICA FIX denoising pipeline, which combines FSL's MELODIC with a more complex automated noise identifier ("FIX"), handled removal of artifacts (e.g., rigid/physiological motion-related) which had survived the Generic fMRI Volume Preprocessing step). Finally, the cleaned functional data were precisely aligned across participants through multimodal surface matching (MSM) registration.

Normalization	see data preprocessing above
Normalization template	MNI standard
Noise and artifact removal	see data preprocessing above
Volume censoring	see data preprocessing above

Statistical modeling & inference

Model type and settings	Multivariate (canonical correlation and partial least squares) analyses with 10-fold cross-validation, mediation analysis with bootstrapping
Effect(s) tested	Multivariate relationships of psychopathology with biological sex, physiological age, self-reported pubertal development, pubertal hormones (Figure 2: analysis 1), of regionally specific neural sexual differentiation with the physiology and psychopathology variates from (1) (Figure 2: analysis 2), of the physiology/psychopathology-linked neural sexual differentiation latent variable scores from (2) --projected onto a different sample--with biological sex, body mass index (BMI), financial deprivation, self-reported pubertal development and psychopathology (Figure 2: analysis 4), and of the physiology/psychopathology-linked neural sexual differentiation latent variable from (2) with patterns of neural sexual differentiation tracking with BMI, financial deprivation, self-reported pubertal development and psychopathology (Figure 2: analysis 5). Analyses 1 and 2 were conducted in the ABCD sample. Analyses 4 and 5 were conducted in the HCP-D sample.

Specify type of analysis: ☒ Whole brain ☐ ROI-based ☐ Both

Statistic type for inference (See Eklund et al. 2016)	Non-parametric inference
Correction	No multiple comparisons, hence no corrections necessary

Models & analysis

n/a	Involved in the study
☐	☒ Functional and/or effective connectivity
☒	☐ Graph analysis
☐	☒ Multivariate modeling or predictive analysis

Functional and/or effective connectivity	Pearson's r
Multivariate modeling and predictive analysis	Partial least squares (PLS) and canonical correlation analyses (CCA) with 10-fold cross-validation, as well as serial mediational analysis. These analyses are described in Figure 2. CCA probed the correlation of psychopathology with sex, physiological age, self-reported pubertal development and pubertal hormones in the ABCD (Analysis 1), as well as the correlation of sexual differentiation in brain function, as estimated in the ABCD sample, with sex (Analysis 4 only), psychopathology and factors relevant to physiological aging (body mass index, financial deprivation, self-reported pubertal development) in the HCP-D sample (Analyses 4 and 5). PLS probed the correlation of regionally specific neural sexual differentiation with psychopathology, physiological age, self-reported pubertal development and pubertal hormones (Analysis 2). The serial mediation analysis (Analysis 3) tested whether the patterns of neural sexual differentiation identified in Analysis 2 (mediator 1) and the physiology CCA variate from Analysis 1 (mediator 2), sequentially mediated the link between polygenic risk for anxiety or depression and the psychopathology CCA variate from Analysis 1. For specificity analysis, we performed a similar mediation analysis anchored in the ADHD PRS.
